# Gaze direction reveals implicit item and source memory in older adults

**DOI:** 10.1371/journal.pone.0226018

**Published:** 2019-12-05

**Authors:** Inés Antón-Méndez, Andrew Talk, Simone Johnston

**Affiliations:** 1 Discipline of Linguistics, School of Humanities, Arts and Social Sciences, University of New England, Armidale, Australia; 2 School of Psychology, University of New England, Armidale, Australia; University of Wisconsin-Milwaukee, UNITED STATES

## Abstract

This study looked at eye movements in relation to source memory in older adults. Participants first studied images of common objects appearing in different quadrants of a screen. After a delay, they were shown cues one at a time presented in all four quadrants. Participants stated whether or not the cue had been seen before and in which location. Participants also rated level of confidence in their responses. In trials where participants either claimed they have not seen a previously presented cue or placed it in an incorrect location, they looked significantly more at the correct quadrant. The proportion of time looking at the correct quadrants during incorrect responses was not related to confidence ratings. These results suggest that eye gaze during the memory task does not reflect memory retrieval below the threshold of verbal report. They instead point to an implicit form of source memory in humans that is accessible to eye movements but not to verbal responses.

## Introduction

Details about the context within which objects and events previously occurred are often important to remember. For example, we may have sense of familiarity with a person we meet, but to appropriately greet them we need to also remember when or where we previously experienced them. The term ‘source memory’ has been used to refer to incidentally acquired memories about one or more components of the context that surrounded a cue when it was previously experienced [1]. Source memories are thus commonly contrasted with memory of the cue itself. Simple behavioral tests have been developed for laboratory study of source memory which involve measurement of memory for details such as the color font in which recognized words were originally presented [2], the gender of the voice in which recognized sentences were originally presented [3], or the area of a computer screen in which a recognized picture was originally presented [4,5].

The cognitive processes of memory have been described by dual-process models that propose involvement of both recollection and familiarity [6,7]. Such models posit that there is a fast and automatic process of familiarity with a previous cue or event which is not accompanied by retrieval of contextual details from the source experience, and only sometimes is there a slower process of recall of information about details of the encoding episode that is the source memory. A wide range of experimental evidence supports dual-process memory models [7,8].

Eye tracking techniques have been extensively used during studies of memory encoding and retrieval. Assessing eye movements and gaze direction on a trial-by-trial basis during encoding provides information about memory processes not available in studies that assess only explicit responses like verbal replies [9]. For example, the amount of fixation on particular visual cues during encoding correlates with subsequent memory retrieval performance [10,11], suggesting that attention to cues is important for encoding memories about them. These findings converge with evidence from studies that do not involve eye-tracking. For example, dividing attention during the encoding session for particular items leads to reduced source memory for those items [12]. The use of items or contexts that have emotional valence to enhance arousal and attention to particular items can also enhance later source recall [2]. Attention to auditory cues in a rodent analog of a source memory task has been studied by indexing attention using head-direction orienting responses rather than by eye tracking. When attention to cues was disrupted by lesion or inactivation of the frontal cortex, reduced source memory about those cues occurred [13,14].

Tracking gaze direction during tests of memory retrieval can also be informative. Studies regularly report a greater number of fixations associated with the correct response even when an incorrect response, or a statement of no memory, is explicitly made [10,15–17]. For example, Ryan et al. [18] measured gaze direction of participants as they viewed scenes, some new, some previously seen and some of which they had previously seen but containing altered elements. The participants looked more at the changed regions of scenes even when they reported that the picture had not changed. Further studies have demonstrated that information about the relationship between particular faces and particular background scenes can be expressed through gaze directions, even well in advance of the performance of explicit behavioral responses [15,19]. Gaze direction during episodic memory retrieval has been found to correlate with explicit recall of the original location of items during particular episodes, but in some cases can occur even when participants fail to make correct explicit responses [20].

In a previous study, we investigated the temporal dynamics of eye movements during a source memory procedure in university students [16]. Our goals were to see whether attention to a cue during encoding is related to later memory of the cue and its source, and whether attention to correct source locations can occur as subjects successfully retrieve a source memory, cannot retrieve a source memory, or retrieve a false source memory. Gaze direction on a trial-by-trial basis allowed us to study how differences in encoding-related eye movements predict subsequent memory, and to examine how eye movements during the test phase characterize successful memory retrieval. We found that extra attention was paid to the correct source location even when participants provided incorrect explicit location responses or provided responses indicating they could not remember the location. Our interpretation of this finding was that, even though the verbal response associated with retrieval of source memory may be dichotomized as correct or incorrect, the underlying processes of source memory are graded. The results of that study are compatible with the hypothesis that implicit memory processes were responsible for the eye movements to the correct source location during trials in which there were incorrect verbal responses. However, those results are also compatible with the hypothesis that participants experienced explicit and conscious partial source recollections and then screened them out in verbal reports, or provided incorrect responses, depending on their confidence (see also [21,22]). The eye gaze responses, being graded, then reflected these different degrees of confidence.

Here we have conducted a further eye-tracking study of source memory to disentangle these two possible interpretations of the relation between eye gaze and memory processes. In the current study we asked the participants to provide confidence ratings for their responses. Confidence ratings are generally assumed to depend on memory processes that are accessible to consciousness (e.g., [17,23]). This allowed a graded verbal response that could then be analyzed for a potential correlation with the graded eye gaze responses. If participants experience explicit and conscious partial source recollections and then screen them out in verbal reports depending on their confidence, then the verbally-expressed confidence judgment data should correlate with the graded eye-tracking data on a trial by trial basis across trials in which participants answered the source memory question correctly. Crucially, on trials in which they were incorrect, there should only be a correlation between gaze and explicit responses if gaze direction is based on the same explicit memory mechanisms that guide the verbal response. A failure to find this correlation would suggest that an independent and implicit source memory process exists that directs eye movements to the correct source location even during trials in which there are incorrect verbal responses. To try to increase the range of source memory performance and source memory confidence levels that could then be correlated with the gaze direction responses, we invited the participation of residents of retirement communities who were older. Source memory performance declines with age, but with a high degree of individual differences [5]. Thus, we expected this population to provide more incorrect response data.

## Materials and methods

This study was approved by the Human Research Ethics Committee of the University of New England. Approval number: HE17-241. Participants provided written consent to participate in this research.

### Participants

27 self-declared healthy individuals over the age of 55 volunteered to participate in the experiment. Of these, 3 were excluded due to the eye-tracker not being able to generate sufficient data to track gaze direction (i.e., more than 50% of the samples taken were invalid; average tracking loss for the remaining participants was 23.69%, ranging from 5.83 to 44.52%). In the remaining 24 participants, there were 12 females and 12 males whose ages ranged from 55 to 86 years (*M* = 69.9).

### Materials

Materials consisted of 96 colored images of common objects which had been developed and described by Cansino et al [4]. From this image pool, a set of 64 images was used for the encoding phase. Thirty-two of the images from the encoding phase were used again in the retrieval phase, together with 32 new images. The same set of images was presented to each participant. The stimuli were presented with E-Prime software (version 2.0) [24] on a laptop computer (1366 × 768 pixels; 60 Hz). Eye movements were tracked by means of a Tobii Technology X2-30 eye tracker situated below the screen. The subjects were seated so that their eyes were 50–80 cm from the screen.

### Procedure

Participants first read and signed an informed consent form and filled out a demographic questionnaire on the computer. This was followed by the experiment proper, which was conducted in a single session consisting of an encoding phase, a retention interval, and a retrieval test phase. Eye movements were tracked during the encoding phase and the test phase.

The experiment commenced with a calibration of the eye-tracking software. Once the calibrations were satisfactory, the encoding phase began. The initial slide served as practice, and this was followed by a series of 32 additional slides. The slides were divided by a red cross into four equal quadrants, two of which (always diagonally opposed) were occupied by two randomly selected pairs of objects. Participants were told to try to remember the objects, as their memory would be tested later, but they were not instructed to remember either object locations or pairings. Only one of the objects from each slide, the target, would later be part of the retrieval phase, while the other (the distractor) would not be seen again. Each participant saw an equal number of cues in each of the four quadrants, but the screen position of each object was counterbalanced across participants. The distractors and targets were the same for all participants. The slides were presented in an individually randomized order. Each trial started with a fixation cross for 1000 ms to center the participant’s focus, followed by the slide for 3000 ms, after which the slide disappeared and the message “press space to continue” appeared. This allowed participants to proceed at their own pace. On average, participants took 916 ms to continue onto the next slide. The whole encoding phase lasted approximately 5 minutes.

**Fig 1:**
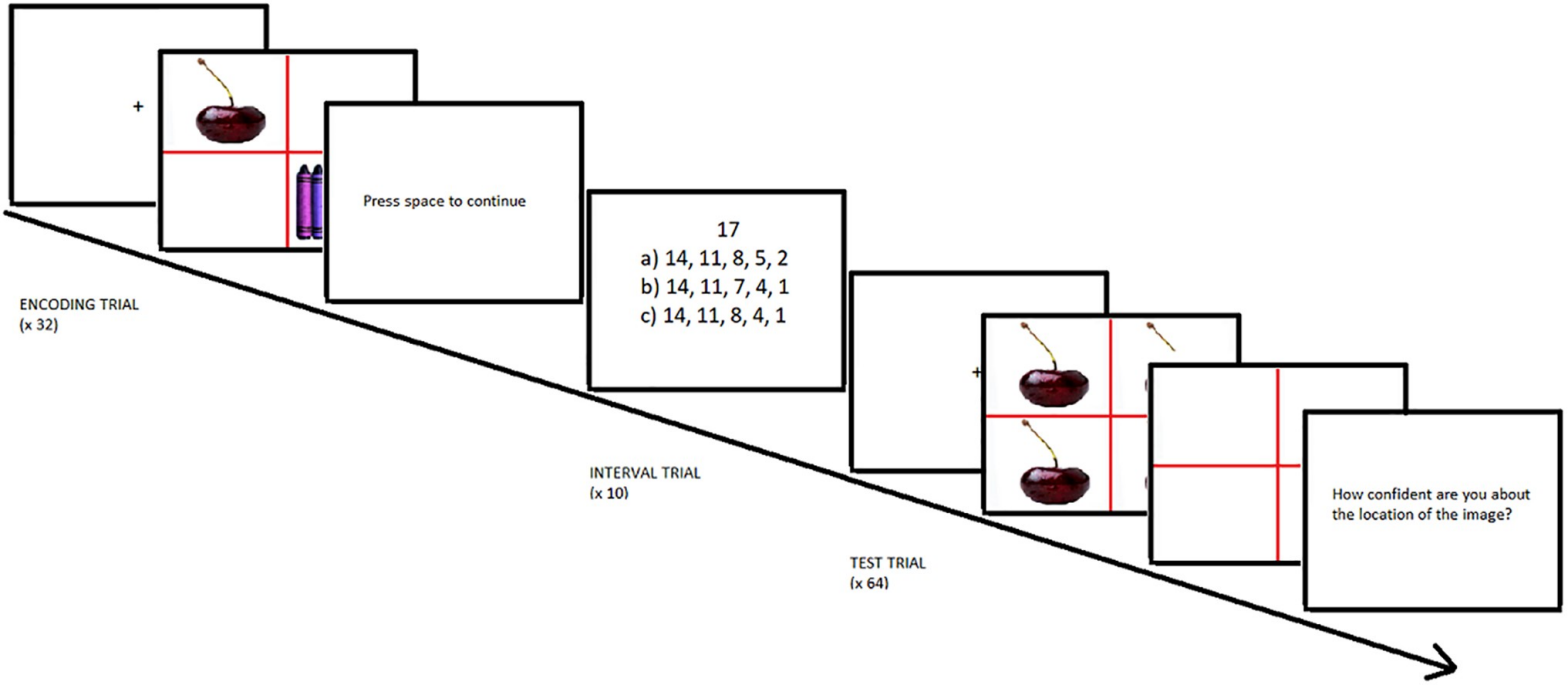
Sequence of events during the session. The figure shows examples of the types of screens encountered by participants in the encoding phase, retention interval and test phase. Please see Methods section for specifics of the timing and participant instructions.

The encoding phase was followed by a ten minute retention interval, where participants performed a filler task that involved counting down by threes from numbers between 17 and 32 (*N* = 10). Each number was presented together with three potential count-down series for the participant to select the correct one (see Fig 1 for an example).

During the test phase, participants were again presented with slides divided into four equal quadrants by a red cross. Unlike the encoding phase, here, each quadrant displayed the same object picture. Participants were asked to look at the objects in each slide to later tell the experimenter whether they remembered it from the previous phase or whether it was "new". If they remembered it, they would be asked to point with their hand to the quadrant it had previously occupied (each quadrant having the same probability of being the correct one). Finally, they were asked to report their level of confidence in their quadrant responses (“How confident are you about the location of the image?”) on a scale from 1 (not sure at all) to 9 (completely sure). If they reported not having seen the object before, they were told to instead rate their confidence that the item was new. After the confidence in the response had been entered, the next trial was automatically initiated.

The test trials began with a fixation cross in the center of the screen for 1000 ms to center the participant’s focus. This was followed by the test slides, which remained on the screen for 3000 ms. Afterwards, the objects disappeared but the red dividing cross separating the four quadrants remained. It was at that time that the verbal responses from the participant were collected and recorded by the experimenter. An example trial can be seen in Fig 1, which depicts the sequence of events for the whole of the session. There were three practice slides at the beginning of the test phase, followed by 64 test slides, 32 of which contained already seen cues. The other 32 contained new cues. The order of presentation of the slides was individually randomized.

### Coding of responses

The third author recorded participants’ responses and later coded these responses according to the following guidelines:

- Incorrect cue recognition–previously seen cues which were labelled as new by the participant.
- Correct cue recognition–previously seen cues which were labelled as previously seen by the participant.
- Correct source location–previously seen cues which were recognized and for which the participant had pointed to the correct quadrant.
- Incorrect source location–previously seen cues which were recognized but for which the participant had pointed to an incorrect quadrant.

New cues could also be correctly identified as new or be falsely recognized. These were not analyzed further.

### Data analysis

The sampling frequency of the eye tracker was 30 Hz, i.e., it took one sample once every 33.33 ms on average. The samples were automatically coded as gaze-detection within any of the four quadrants, or as no detection, which could be due to the participant looking outside the areas of interest or to a failure to detect gaze direction (as can happen, for example, during blinks). For data collected during encoding, the averaged number of gaze samples falling within the target or distractor quadrant across the entire trial was calculated. For data collected during retrieval, the number of gaze samples falling on the target or any of the competitor quadrants was calculated for each 100 ms time bin (since there were three competitor quadrants (one of which was sometimes selected) and only one target, the number of samples directed to competitors was averaged according to the number of not selected competitors–three in correct responses, and two in incorrect source responses).

Several types of analyses were carried out. Linear mixed-effects logistic regression (LME) [25] using the lme4 package in R version 3.4.4 [26,27] was employed to see the effect that initial time spent looking at a cue during encoding had on the likelihood that the cue and the source would later be correctly identified. The models modelled the number of samples in which gaze was detected as falling on a given quadrant on a given trial as a function of the fixed factors (predictors) of quadrant content (target or distractor) and response accuracy (correct or incorrect cue recognition, or source location). A maximal random effect structure including random intercepts and slopes over subjects and items was always attempted first [28]. However, failure to converge of the model for source memory required a simplification of the random effects which was implemented by reducing the item random effect to include only the intercept [28].

In the test phase, rather than total number of detections, it was important to consider the dynamics of eye movements across time since differences in the timing of fixations could be key to distinguish between recognition and random inspection. For this, growth curve analyses (GCA) [29] were carried out in R. The models modelled the number of samples in which gaze was detected as falling on a given quadrant at different 100 ms time bins from the onset of the test slide as a function of two predictors: quadrant content (whether it was the quadrant where the target had been previously presented or one of the competitor quadrants), and whether the quadrant figured in the participants verbal response. The overall time course in the GCAs was modelled with a first (linear), a second (quadratic), and a third (cubic) orthogonal polynomial. The models included a maximal random effect structure consisting of random effects of the three time terms over subjects and the linear and quadratic time terms over subject-by-quadrant content. In these analyses, a significant intercept for a given main effect or interaction indicates an overall difference of total gaze detections associated with different quadrants due to that main effect or interaction–i.e., whether there were differences in the total number of gaze detections for different quadrants regardless of how these gaze detections were distributed across time. An effect of the linear term gives an indication of whether the number of gaze detections rose or fell differently according to the main effect or interaction–that is, whether there were differences in the slopes associated with looks to different quadrants. Finally, an effect of the quadratic or cubic terms indicates differences in the shape of the curve across the time window suggesting more complex differences in the dynamics of eye movements associated with different quadrants as a result of the main effect or interaction [16].

Lastly, to see whether the confidence ratings could explain participants’ behavior, LME was used to look at differences in relation to the response. Response type was modelled on the basis of confidence level as a fixed factor, including independent random factors of slopes over subjects and items, and confidence levels over subjects and items. Additionally, Pearson correlation was used to analyze the relationship between gaze detections and confidence levels for each of the different response types. This was done by first averaging the proportion of target viewing time (i.e., gaze detections on the target in relation to total gaze detections in the trial) in all the trials within a given response type and associated with a given confidence level for each participant; and then correlating that measure of target viewing time with confidence level.

## Results

### Task performance

The percent of previously seen cues that were correctly recognized was 81.0 ± 3.5 (mean ± *S*.*E*.), leaving 19.0 ± 3.5 of previously seen cues not recognized. Of previously seen cues correctly recognized, 61.5 ± 4.3 locations were accurately indicated, and 38.5 ± 4.3 locations were incorrectly indicated. The percent of new cues correctly identified as new was 77.3 ± 3.6.

When participants selected a wrong source location, they tended to choose a quadrant adjacent to the correct quadrant in preference to the diagonally opposite quadrant. In such trials, the adjacent quadrants in the horizontal direction were chosen 32% of the time, adjacent quadrants in the vertical direction were chosen 43%, and the opposite quadrant was chosen 25% of the time. There seemed to be a small bias for choosing the top left quadrant (28% of the time vs. 23–25% for the other quadrants). This, however, does not affect the analyses below as quadrant position was counterbalanced–all cues appeared in all positions (in different lists), and subjects saw equal numbers of cues in each of the positions.

### Eye movements during the encoding phase

**Fig 2:**
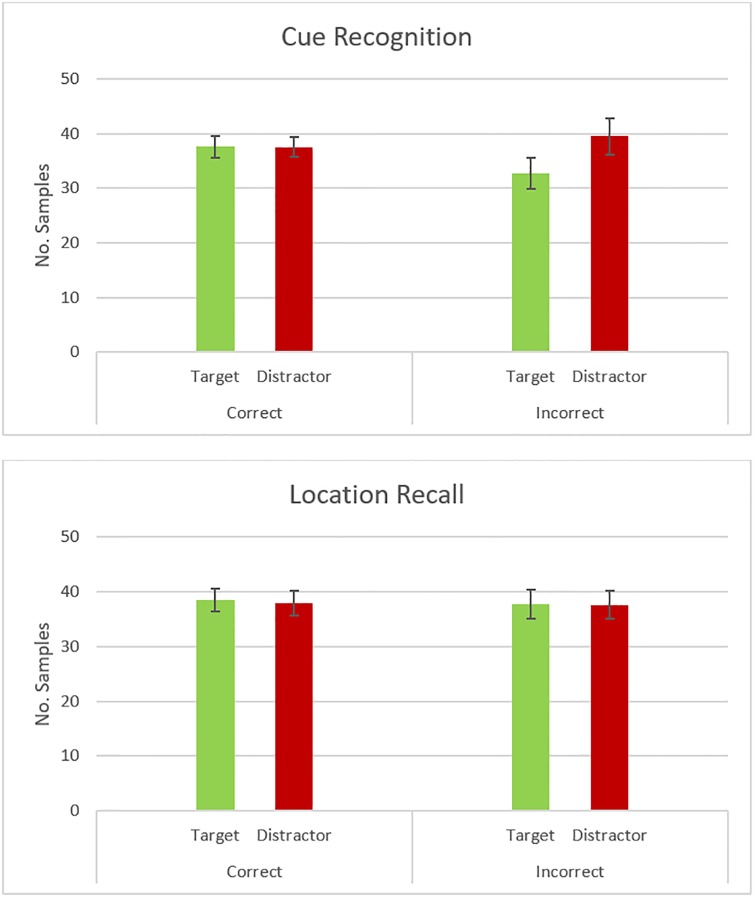
Attention to cues during encoding. The amount of time participants looked at the target or distractor cue during the encoding phase was not related to whether they would later recognize the cue or remember the source location of the cue. The graph shows the number of eye-tracking samples (per trial/per subject) in which gaze was detected as being on the target or distractor cue (and *S*.*E*. of the mean). Data are divided according to whether the cue was later recognized or not (top panel) and, if recognized, whether its source location was correctly remembered (lower panel).

Fig 2 shows the average number of gaze detections falling on the target quadrants (i.e., the quadrants with the cue that would be presented later in the retrieval phase) and distractor quadrants (i.e., the quadrants with the cue that would not be seen again) per trial per person during the whole three seconds of the encoding phase. The top panel shows numbers of gaze detection samples according to later cue recognition accuracy, and the bottom panel shows number of such samples according to later source location accuracy.

A comparison of gaze detections within different quadrants in each trial (*N* = 768) according to their content (target vs distractor) and whether the target cue was later recognized did not result in statistically reliable differences between targets and distractors overall (*Quadrant content estimate* = -3.20, *SE* = 3.17, *p* = .321), or between trials in which the cue was later correctly recognized vs when it was not recognized (*Cue recognition estimate* = -1.53, *SE* = 1.77, *p* = .390). The interaction between these two factors was also not statistically significant (*Interaction between quadrant content and cue recognition estimate* = 3.17, *SE* = 2.85, *p* = .279).

Analyzing gaze detections within different quadrants as a function of quadrant content and whether the location had been correctly identified or not (excluding trials where the cue had not been correctly recognized; *N* = 622) again failed to show an effect of quadrant content (*Quadrant content estimate* = -0.24, *SE* = 2.76, *p* = .932), an effect of source memory (*Source memory estimate* = 1.27, *SE* = 1.90, *p* = .507), or an interaction between the two (*Interaction between quadrant content and source memory estimate* = 0.01, *SE* = 3.17, *p* = .998).

### Eye movements during the test phase

**Fig 3:**
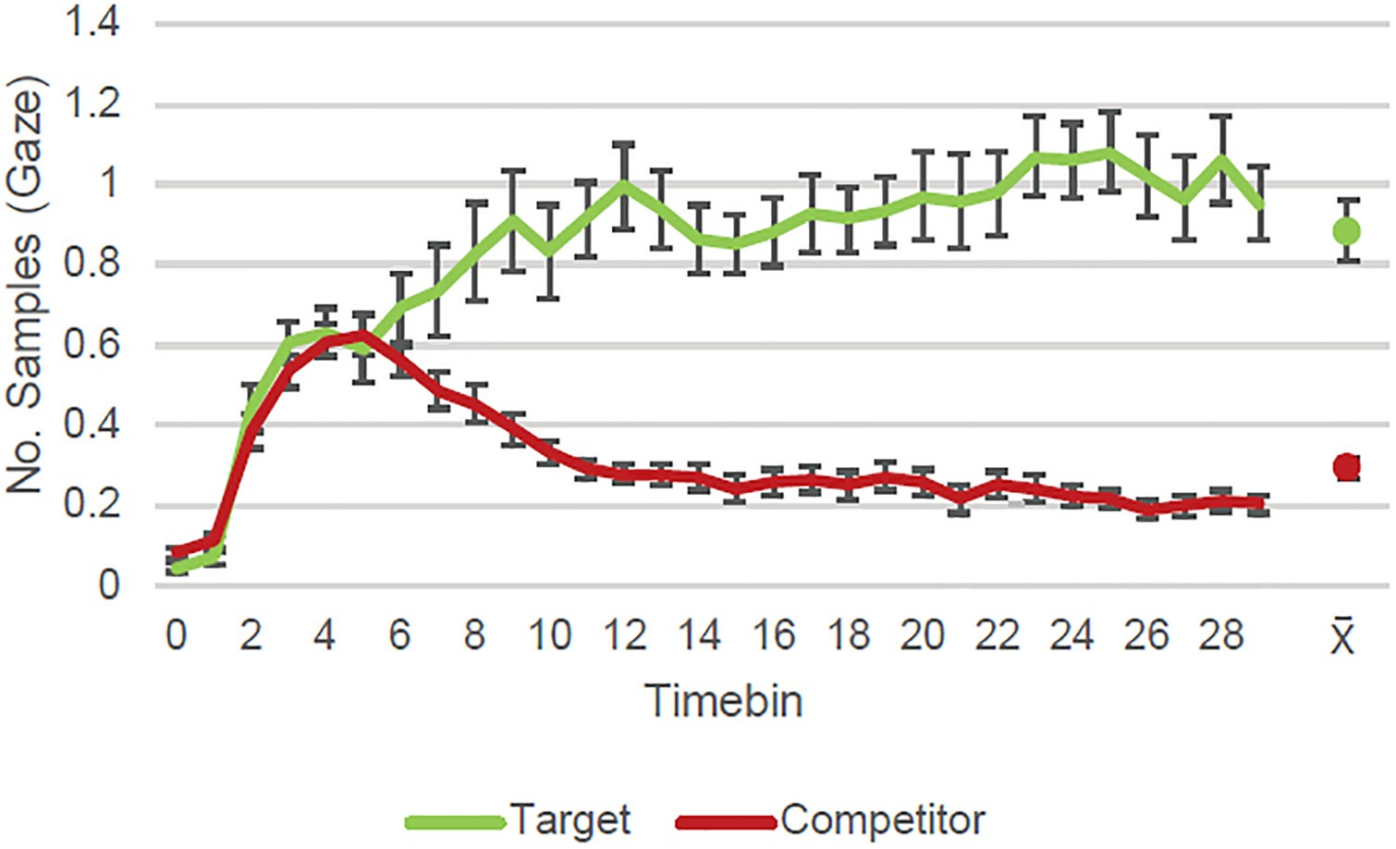
Correct recognition and source responses. In the time window of interest (starting at timebin 5; 500 ms after stimulus onset), participants looked more to the correct target quadrant than to the competitors when they recognized the cue and correctly identified its source location. The graph shows eye-tracking samples (per trial/per subject) with gaze detected (and *S*.*E*. of the mean) during retrieval according to quadrant content for trials with correct source memory. Data aggregated on 100 ms time bins. The dots represent each line’s average (and *S*.*E*.) across the time window of interest (timebins 5–29).

**Fig 4:**
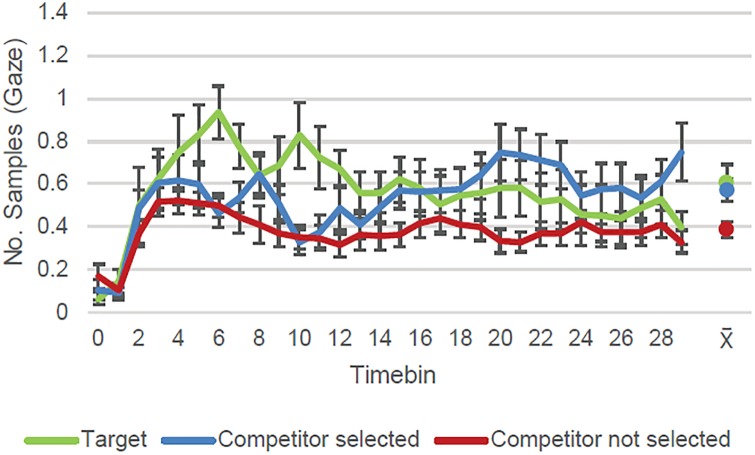
Correct recognition but incorrect source responses. In the time window of interest (starting at timebin 5; 500 ms after stimulus onset), participants looked more to the correct target quadrant than to the competitors not selected when they recognized the cue but incorrectly identified its source location. The graph shows eye-tracking samples (per trial/per subject) with gaze detected (and *S*.*E*. of the mean) during retrieval according to quadrant content for trials with incorrect source memory. Data aggregated on 100 ms time bins. The dots represent each line’s average (and *S*.*E*.) across the time window of interest (timebins 5–29).

**Fig 5:**
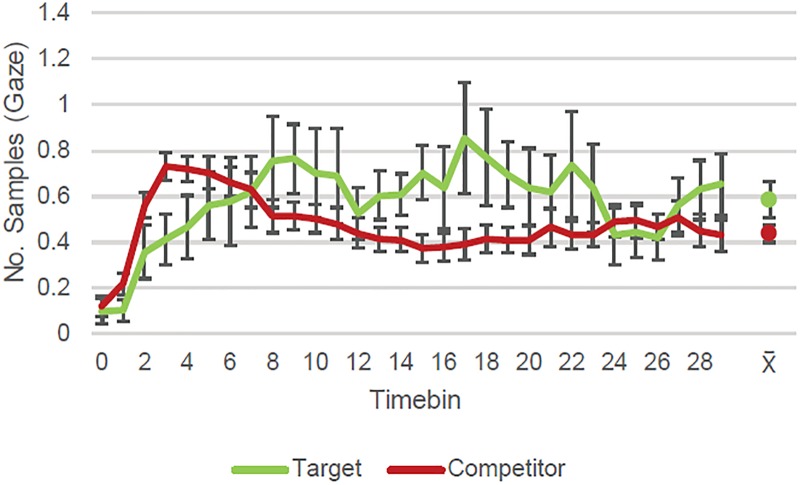
Failed recognition. In the time window of interest (starting at timebin 5; 500 ms after stimulus onset), participants looked more to the correct target quadrant than to the competitors when they failed to recognize the cue and incorrectly labeled it as being new. The graph shows eye tracking samples (per trial/per subject) with gaze detected (and *S*.*E*. of the mean) during retrieval according to quadrant content for trials with failed cue recognition. Data aggregated on 100 ms time bins. The dots represent each line’s average (and *S*.*E*.) across the time window of interest (timebins 5–29).

**Fig 6:**
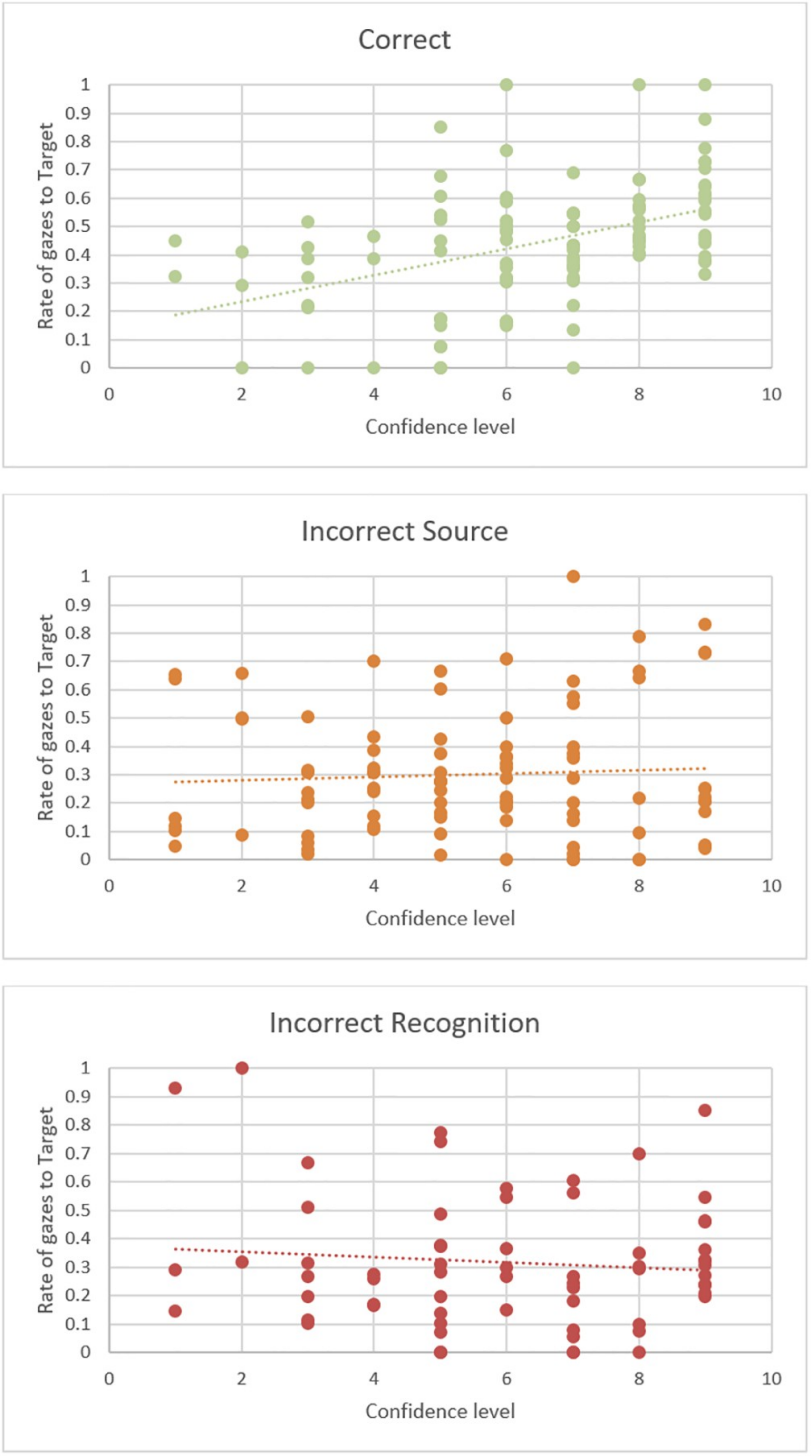
Relationship between confidence and gaze. When participants correctly identified the source location of a test cue, their reported confidence level correlated with time spent looking at the target. This relationship did not occur for test trials in which an incorrect response occurred. The graphs show the proportion of gaze samples falling on the target quadrant (averaged across relevant trials for each participant) for each of the response types as a function of reported confidence level in the location response (for Correct and Incorrect Source responses) or in the ‘new’ response (for Failed Recognition responses).

Figs 3–5 show the number of samples in which gaze was detected as falling on the target and competitor quadrants within 100 ms time bins per trial per person in the test phase. Each figure shows the dynamics of the eye movements for different subsets of the trials depending on the accuracy of the verbal responses. We chose the time windows of interest on the basis of results obtained on a similar task with younger participants [16]. Data from that experiment suggested the existence of two distinct time windows (see [16] Figs 3–6): an initial time window from the time the slide came into view until around 500 ms, during which interval participants seem to perform a quick inspection of all quadrants; and a second window from 500 ms until the slide disappeared (3000 ms) in which participants showed looking preferences for one or two of the quadrants. This pattern is also evident in the data for the current cohort (see Figs 3–5). In consequence, we again focused our analyses on the interval between 500 ms and 3000 ms.

For trials in which the cue’s source had been correctly indicated (*N* = 405; Fig 3), the GCA (due to non-convergence of the full model, the random effect structure was reduced by removing random effect of the quadratic term over subject-by-quadrant content) showed the number of gaze detections within different quadrants to be dependent on quadrant content (target vs. competitor) both with respect to total number of gaze detections, greater for target quadrants (*Quadrant content intercept estimate* = 0.36, *SE* = 0.03, *p* < .001), and with respect to the dynamics of eye movements across time (*Interaction of quadrant content with linear term estimate* = 0.55, *SE* = 0.11, *p* < .001; *Interaction of quadrant content with quadratic term estimate* = -0.30, *SE* = 0.03, *p* < .001; *Interaction of quadrant content with cubic term estimate* = 0.13, *SE* = 0.03, *p* < .001). This indicates that, as expected, the target quadrant which the participant intended to name in their response was looked at more often than the other quadrants on the slide overall, looks converged on this quadrant faster, and it was then preferentially fixated.

We also analyzed the eye movement patterns during the test phase for trials where one of the competitors had been wrongly selected (i.e. incorrect source memory trials; *N* = 226; Fig 4). The target quadrant and the competitor selected were looked at significantly more often overall than the competitors not selected (respectively, *Target-Competitor not selected intercept estimate* = 0.10, *SE* = 0.03, *p* = .001; *Competitor selected-Competitor not selected intercept estimate* = 0.09, *SE* = 0.03, *p* = .001). There were no statistically reliable differences in amount of time participants looked at the correct target coordinate and the quadrant they wrongly selected (*Target-Competitor selected intercept estimate* < 0.01, *SE* = 0.03, *p* = .920).

As for the temporal dynamics of the eye movements, the target was significantly different on the linear term both from the competitors not selected (*Interaction with linear term estimate* = -0.21, *SE* = 0.11, *p* = .050; *Interaction with quadratic term estimate* = -0.01, *SE* = 0.09, *p* = .869; *Interaction with cubic term estimate* = 0.06, *SE* = 0.05, *p* = .190), and from the competitor selected (*Interaction with linear term estimate* = -0.33, *SE* = 0.11, *p* = .003; *Interaction with quadratic term estimate* = -0.03, *SE* = 0.09, *p* = .711; *Interaction with cubic term estimate* < 0.01, *SE* = 0.05, *p* = .840). The two types of competitors were not significantly different from each other on any of the time terms (*Interaction with linear term estimate* = 0.11, *SE* = 0.11, *p* = .282; *Interaction with quadratic term estimate* = 0.02, *SE* = 0.09, *p* = .838; *Interaction with cubic term estimate* = 0.06, *SE* = 0.05, *p* = .191). In sum, the target quadrant attracted looks more quickly than the competitors and was associated with a different pattern of eye movements from both of them (selected and not selected competitors) across the time window. On the other hand, the two types of competitors did not differ in *how* they were inspected (although, as already noted, they did differ in *how much* they were looked at).

Finally, we analyzed the time course of gaze detections within target and competitor quadrants for those trials in which the previously viewed cue was not recognized and was incorrectly declared as being new (*N* = 169; Fig 5). Participants looked significantly more often at the target quadrant than the competitors (*Quadrant content intercept estimate* = 0.08, *SE* = 0.03, *p* = .007), and the dynamics of the eye movements were also different as reflected by a significant difference on the cubic term, which signals a difference in the inflection points (S-shape) of the two curves (*Interaction with linear term estimate* = 0.02, *SE* = 0.13, *p* = .902; *Interaction with quadratic term estimate* = -0.26, *SE* = 0.15, *p* = .088; *Interaction with cubic term estimate* = 0.19, *SE* = 0.05, *p* < .001).

### Confidence judgments during the test phase

The participants reported the level of confidence in their location responses when they selected a quadrant or, otherwise, in their ‘new’ responses. These confidence judgments differed between the response types (*Confidence estimate* = -0.38, *SE* = 0.06, *p* < .001) in that participants expressed more confidence in their correct responses (*M* = 7.6, *S*.*D*. = 0.9), than in their incorrect ones (incorrect source memory responses *M* = 6.1, *S*.*D*. = 1.7; failed recognition responses *M* = 6.6, *S*.*D*. = 1.5). For correct responses, there was a significant positive correlation between confidence level and the proportion of time spent looking at the target quadrant as opposed to the competitor quadrants (*r* = .45, *p* < .001; see Fig 6). For incorrect source memory responses, and failed recognition responses, there was no statistically reliable relationship (incorrect source memory *r* = .05, *p* = .304; failed recognition *r* = -.10, *p* = .220).

## Discussion

Eye movements have been shown to accompany memory processes, and to be fast and automatic [9]. Here, we assessed gaze direction on a trial-by-trial basis during the encoding and retrieval phases of a source memory task, as well as asking participants to report on their recollection of presented cues. We studied whether attention to cues appearing on a computer screen predicts later recognition and memory for the prior screen location (i.e. the source) of the cue. Also, during the test phase, we studied whether participants attended to the original screen location of the cue during successful and unsuccessful cue recognition and source memory retrieval. Finally, we asked participants to provide ratings of confidence in their location responses to determine whether a continuous model for source retrieval is better related to gaze direction during the test than a dichotomous model based on correct or incorrect explicit performance, and whether eye movements are likely to reflect implicit memory processes.

### Attention to cues during memory encoding

We found that the degree of attention participants allocated to a cue during the encoding phase did not have an effect on later recognition of the cue or on memory for the cues’ original screen location. There were no statistically reliable overall differences between the amount of time targets and distractors were fixated during the encoding phase, and no relationship between the amount of time spent looking at a cue during encoding and later recognition and source memory accuracy. This is consistent with results of our previous study employing a very similar procedure [16]. On the other hand, as already mentioned in the introduction, several studies have found that the amount of attention paid to visual cues during encoding affects subsequent memory performance for those items [2,10–12,30,31]. What is more, given the role the prefrontal cortex plays in attention [32,33], the dependence of memory strength on attention is supported by reports of the impact on memory of frontal cortical lesions both in humans [34] and animals [35]. All this is unsurprising as, naturally, worse encoding due to reduced attention during study should result in weaker memory traces and impaired memory test performance [10]. Yet, with the current task, we do not find a significant difference in the time spent looking at the target at encoding in relation with subsequent response accuracy. It is possible that the current experimental design did not allow such effects to surface. Here, participants had ample time (three seconds) to look at the target and distractor cues in the encoding trials and, in contrast to Kafkas and Montaldi’s [30] or Olsen et al’s [31] studies which looked at incidental encoding, they were specifically asked to try to remember the objects. This may have resulted in sufficient attentional investment to be devoted to both of the cues at study, therefore preventing the required differences to arise later. Additionally, while our study only required participants to attend to 32 items, Kafkas and Montaldi’s contained 120 items and, in consequence, it would have made it more likely for participants’ attention to wax and wane. In other words, perhaps the effect only appears when the task allows for more substantial differences in visual attention during encoding.

The fact that variability in recall can arise in the absence of variability in attention allocation at encoding could mean that mechanisms other than poor encoding [10] are responsible for differences between recalled and not recalled cues in this case (e.g., proactive interference [36,37]). It is also known that older adults are more prone to poor encoding generally [38], and this may not be always related to attention allocation [39].

### Attention to quadrants during test

Despite expectations of this elder cohort of participants incurring substantial numbers of incorrect responses, verbal reports were mostly accurate regarding recognition of previously seen cues (81.0% of old cues were correctly recognized; in contrast with 22.7% of new cues falsely recognized), and above chance regarding recall of the original location in which the cue was presented (49.8% of all cues were correctly located, in contrast with a chance percentage of 25%). Even when participants did not provide an accurate verbal response, their gaze was more likely to fall on the screen quadrant where the cue had been originally presented than on competing quadrants. More specifically, when they claimed to have recognised the cue but not remembered its source, participants were just as likely to look at the correct location as at the incorrectly chosen location and significantly more than at the other locations (Fig 4). Remarkably, they were also more likely to look at the correct quadrant than any of the other ones when they reported not recognising the cue (Fig 5). Finally, our participants rated their confidence in their location responses if they selected one of the quadrants or in their not having encountered the cue before if they reported it was new. They were more confident when they were correct and, within correct responses, the more confident they were in their response, the longer they looked at the target. In contrast, for incorrect responses, no correlation was found between confidence level and time looking at the correct quadrant.

The discrepancy between the knowledge implied by the verbal responses and that implied by the pattern of eye movements is consistent with data from younger participants on a very similar task [16], who also looked longer at the target location than at competitors not selected when they had incorrectly located the recognized cue. One explanation for this behavior is that verbal responses of recollection, including memory of source, necessarily dichotomize underlying processes of memory retrieval that are likely to be graded [16]. The question is whether looking preferences reflect conscious uncertainty or implicit memory without awareness.

Evidence of recognition without awareness has been found in several studies of forced-choice responses in which pure guesses resulted in significantly higher accuracy than would have been predicted by chance alone [40–43]. However, those results apply to memory for the target cues themselves, not of the sort of ancillary contextual details that constitute source memory. In fact, Craik et al [40] suggest that the difference between implicit and explicit recognition is that “explicit memory is accompanied additionally by some representation of the context of initial occurrence” which can be more or less specific, further differentiating between full recall and a feeling of knowing or familiarity [40]. In contrast, participants in the current study and in Talk et al [16] show signs of remembering one aspect of the context, namely location, when their explicit responses about this same aspect were mistaken. It could thus be that they have retrieved the memory, but the context representation is weaker, inducing more conscious competition between alternative quadrants as the possible correct response. This in turn would result in gazes being directed to the two alternative locations.

However, the fact that there is no correlation between self-reported confidence levels for incorrect responses and gazes to the correct target location suggests otherwise. If gazes directed to the target location were a reflection of the participant being consciously unsure of the original location, we would have expected participants to look more towards the correct location the less confident they were in the eventual incorrect response given. Instead, gazes to the correct location for incorrect location responses were equally prevalent regardless of the participant’s uncertainty about their response. Furthermore, the participants in the current study also evidenced memory of source when they failed to recognize a cue. Therefore, for incorrect responses, eye movements must have been a reflection of participants’ implicit memory of the cue *and* its source. This interpretation is supported by results by Laeng and colleagues on patients with amnesia due to hippocampal lesions [43] who were also unable to explicitly recollect previously seen items but whose eye movements showed implicit memory of the item’s original location. This contrasts with the results of Urgolites et al [17] on similar patients who did not show preferential looking to previously seen items in trials for which there was no evidence of explicit recall (e.g., low confidence ratings). We believe that the conflicting evidence is an artefact of the way the analyses were carried out. Urgolite et al’s ‘preferential viewing’ is an overall measure across the entirety of the time window (3 s) which would not be able to capture the differences in eye movement dynamics that we find in our time-based analyses. For example, in Fig 4, if we had only considered overall looking times to target and selected distractor, we would also have failed to find any preferential viewing for the target in relation to the chosen distractor (see [19] in relation to the higher sensitivity of time-course measures).

The existence of implicit source memory argues against Craik et al’s [40] suggestion that what differentiates explicit and implicit recognition is the presence of some representation of the original occurrence’s context. It also argues against source memory signalling episodic memory [44,45] given the latter’s association to conscious recollection [46,47]. Instead, memory of source appears to also be based on some kind of graded process. While it is tempting to think of this graded source memory process as being related to familiarity, this is unlikely as familiarity only appears to contribute to source memory in very specific circumstances, i.e., those that make the degree of familiarity of an item a clue to its source [8,48], which this design does not seem to meet. Furthermore, graded memory processes are not restricted to familiarity [49]. In truth, the current design does not allow us to extract conclusions as to the nature of this graded source memory.

### The age factor

In line with findings that source memory worsens with age [5,50], our older participants had slightly lower recognition and source location scores than those in Talk et al [16] who were on average 50 years younger: cue recognition differed by 2%, and source memory by about 5% (correct recognition for the younger cohort was 83.2% on average, and correct source memory was 66.4%; in contrast with 81.0% and 61.5% respectively for the older cohort). If false alarms are taken into account, the difference between the two groups in terms of recognition becomes starker as the older participants were more likely to incorrectly declare a new item old (mean d-prime for older cohort = 2.10 vs mean d-prime for younger cohort = 3.16). Notice that, even though the false alarm rate was higher for the older cohort, this cannot mean that they had a lower response threshold since their overall hit rate was still lower than that of the younger cohort. At this point, we can only speculate about the reasons for this difference, but one possibility is that the older population is more susceptible to interference [51]. There were also differences between the two populations in terms of eye movements. In incorrect location trials (after correct recognition), the older participants looked as much to the target as to the competitor selected, while the younger participants looked more to the competitor selected than to the target. Additionally, while the older participants also showed a looking preference for the target quadrant even when they failed to recognize the cue, the younger participants of Talk et al [16] did not. In sum, the explicit verbal responses of the younger cohort are more accurate than those of the older cohort, but the looking patterns of the older cohort in inaccurate responses seem to show stronger implicit memory than those of the younger cohort.

In the absence of data allowing for a direct comparison, it is not possible to say with certainty what could be the reason for these differences. One speculative explanation rests on differences in response criteria. Shifts in response criterion have been found to occur with age [51], as well as in response to differing task characteristics [40], and also vary across individuals [52]. If the younger population’s verbal responses were based on a laxer criterion–i.e., a weaker match between the cue and the memory trace was sufficient for this population to respond positively, there would be a number of trials in which the strength of the match between cue and memory trace is equivalent for younger and older participants, but which would lead to correct responses for the younger population and incorrect responses for the older cohort. The eye movements would then be reflecting the (not acted upon) match resulting in the target being fixated more often in incorrect responses for the older population than the younger population. It would be worth investigating this in the future as, were this explanation to prove true, accounts of memory decline with age that rely on verbal responses could sometimes be overestimating the actual memory loss due to age. Instead, it could be that, for some individuals, the decline could have more to do with variations in response criterion than cognitive deterioration.

Another possible reason for the apparent differences between the two cohorts (suggested by an anonymous reviewer) may have to do with timing. Older adults may take longer to make a decision and this may be reflected in their eye movements. This should then result in similar looking patterns as the younger adults but starting later in the time window, which is what appears to be happening in the case of incorrect source responses–older adults start to look more at the competitor eventually selected at about 1800 ms while younger adults are already primarily looking at it more than a second earlier (Cf. Fig 4 here and Fig 4 of Talk et al. [16]).

## Conclusion

In conclusion, the accumulated evidence seems to point towards gaze being an indicator of both explicit and implicit source memory [9–11,16]. First, gaze direction has been found to be reliably directed, not just towards a previously seen visual cue, but towards the cue in its original context (i.e., source). And second, this happens even in trials in which the response is incorrect, and independently of confidence levels in the incorrect response.

This is compatible with continuous, rather than all-or-none, models of source memory. The graded nature of the memory suggests the existence of a criterion governing response decisions, such that memory traces that reach the criterion level will be explicitly acknowledged, but those that do not will not be considered to correspond to a previously encoded memory. Perhaps it is this criterion which varies between the older participants in the current study and the younger participants of Talk et al [16], explaining the slightly worse recognition and source memory scores of older participants. On the other hand, implicit memory measures suggest older participants are on a par with the younger cohort in terms of memory encoding.
